# Gestational Diabetes Mellitus Does Not Change the Pharmacokinetics and Transplacental Distribution of Fluoxetine and Norfluoxetine Enantiomers

**DOI:** 10.3390/pharmaceutics17010035

**Published:** 2024-12-30

**Authors:** Daniela Miarelli Carvalho, Glauco Henrique Balthazar Nardotto, Gabriela Campos de Oliveira Filgueira, Geraldo Duarte, Ricardo Carvalho Cavalli, Vera Lucia Lanchote, Elaine Christine Dantas Moisés

**Affiliations:** 1 Department of Obstetrics and Gynecology, Ribeirão Preto Medical School, University of São Paulo, Ribeirão Preto 14049-900, SP, Brazil; 2 Department of Clinical Analyses, Toxicology and Food Science, School of Pharmaceutical Sciences of Ribeirão Preto, University of São Paulo, Ribeirão Preto 14040-903, SP, Brazil; 3 Department of Pharmacology & Therapeutics, Roswell Park Comprehensive Cancer Center, Buffalo, NY 14263, USA; 4 Bioanalytics, Metabolomics & Pharmacokinetics Shared Resource, Roswell Park Comprehensive Cancer Center, Buffalo, NY 14263, USA

**Keywords:** fluoxetine, norfluoxetine, enantiomers, pharmacokinetics, pregnancy, placental transfer

## Abstract

**Background/Objectives**: Fluoxetine (FLX) is the inhibitor of serotonin reuptake most prescribed in pregnant women with depression. This study evaluates the influence of gestational diabetes mellitus (GDM) on the enantioselective pharmacokinetics and transplacental distribution of FLX and its metabolite norfluoxetine (norFLX). **Methods**: Ten pregnant women diagnosed with GDM (GDM group) were investigated in the third trimester of gestation after they achieved good glycemic control. They received a single oral dose of 20 mg FLX, and blood samples were collected from 0 to 672 h. On the day of delivery, after another single oral dose of 20 mg FLX, blood samples of maternal vein, umbilical vessels and intervillous space were collected at birth. The pharmacokinetics parameters obtained for pregnant women diagnosed with GDM were compared with a group of healthy pregnant women (n = 9) previously investigated using Kruskal–Wallis’s rank-sum test with the Dunn–Bonferroni post hoc test. **Results**: The area under the plasma over time curve (AUC_0–∞_) were 197.93 and 109.62 ng∙h/mL for FLX and 600.39 and 960.83 ng∙h/mL for norFLX, respectively, for their R-(+)- and S-(-)- enantiomers. The umbilical vein/maternal vein ratio for FLX and norFLX enantiomers was nearly 0.3, inferring low placental transfer. The umbilical artery/umbilical vein ratios were nearly 0.7 for both FLX and norFLX enantiomers, indicating absence or small fetal metabolism. **Conclusions**: The GDM did not alter the pharmacokinetics of FLX and norFLX enantiomers in patients with good glycemic control evaluated in the third trimester of gestation.

## 1. Introduction

The overall prevalence of gestational diabetes mellitus (GDM) is nearly 16% [1,2]. GDM has been considered the most common metabolic problem during pregnancy [3], reaching up to 25% of pregnancies, depending on the diagnostic criteria and aspects related to the population studied, such as ethnicity, genetics, cultural elements, typical diet, and predominant biotype [4].

The most common risk factors for the development of GDM are metabolic syndrome, overweight, age over 35 years, excessive weight gain during pregnancy, excess of body fat, family history of diabetes, fetal overgrowth, polycystic ovary, short stature, previous obstetric history of gestational diabetes mellitus, macrosomic neonate, and perinatal death [4]. A healthy diet and physical activity can prevent GDM and control the blood glucose [3].

Mood disorders are also clinically significant and highly prevalent conditions observed during the gestational period. The prevalence of depression in pregnant women is estimated to be 14–23% [5,6]. The postpartum depression rate is 10–15% [7], but it can reach up to 33%, depending on the places and cultures surveyed and diagnostic criteria [8,9]. One in twenty nonpregnant women in childbearing age experience major depression, of which approximately one in three report antidepressant use [10].

Higher depression was reported in pregnant women with GDM (25.92%) than in those with no GDM (10.38%) [11]. Pregnant women with GDM had a nearly twofold greater risk of being diagnosed with depression compared to those without GDM [1]. Untreated depression causes several adverse effects for both mother and child, the most common being spontaneous abortion, prematurity, low Apgar score, developmental delay, and low birth weight [12].

Fluoxetine (FLX) is the most commonly prescribed inhibitor of serotonin reuptake for the treatment of depression in pregnant women, because it is considered safe and effective; however, it is still advisable to consider the risks with caution [12,13]. FLX is a racemate drug consisting of 50% S-(+)-FLX and 50% R-(-)-FLX enantiomers. Its active metabolite also consist of two enantiomers: S-(+)-NorFLX and (R)-(-)-NorFLX. The two FLX enantiomers have similar serotonin reuptake inhibitor potency, while S-(-)-NorFLX may be up to 20 times more potent than R-(+)-NorFLX [8,14]. FLX has high bioavailability (90%) and plasma protein binding (94.5%) [15,16]. The clearance of S-(+)-FLX and R-(-)-FLX in healthy pregnant women were reported as 0.66 and 1.45 L/h/Kg [17].

It has been observed that diabetes may affect the absorption, distribution, metabolism and excretion of many drugs due to changes in blood flow, gastric emptying, non-enzymatic glycation of albumin, and activities of enzymes and dug transporters, among other factors [18]. Previous reports of our research group showed that controlled type 2 diabetes mellitus in hypertensive pregnant women does not influence the pharmacokinetics of nifedipine, a drug highly bound to plasma protein and with a metabolism dependent on CYP3A [19]. In addition, the enantioselective kinetic disposition of metoprolol, a drug with a metabolism dependent on CYP2D6 and CYP3A, was also not changed in pregnant women diagnosed with GDM and with good glycemic control, except by the prolonged time to reach the maximum plasma concentration (tmax) of the unchanged drug and its main metabolites [20]. However, higher AUC values (162.7 vs. 84.2 ng.h/mL) of (SR)-labetalol, the isomer with α-blocking activity, was observed following oral administration of the racemic drug to pregnant women diagnosed with GDM, but without any observation regarding the glycemic control [21].

Both pregnancy and diabetes may change the pharmacokinetics of drugs by different mechanisms. The changes caused by diabetes on pharmacokinetics are probably linked to hyperglycemia condition [18]; it is not possible to generalize the effect of diabetes on pharmacokinetics of one drug to another, and the effect of GDM on pharmacokinetics remains unclear, and this study evaluates the impact of GDM on the enantioselective pharmacokinetics and transplacental distribution of FLX and its metabolite norFLX in patients with good glycemic control.

## 2. Materials and Methods

### 2.1. Clinical Trial

The clinical study was approved by the Ethics Committee of the local hospital of the Ribeirão Preto Medical School, University of São Paulo (HCFMRP-USP) (Approval No. 14806/2012 on 3 December 2012). All patients were investigated after signing the informed consent form.

The study enrolled 10 pregnant women at the third trimester of gestation (gestational age of 32–34 weeks) previously diagnosed with GDM, according to WHO guidelines [22], only after they had achieved successful metabolic control; besides GDM, they had no other comorbidities and no fetal repercussions. Clinical data of the participants and their newborns were collected.

The GDM diagnostic was made according to international criteria that consider one or more blood glucose values within the following standards: fasting blood glucose (one and two hours after overloading with 75 g of anhydrous glucose, respectively) higher or equal to 92 and less than or equal to 125 mg/dL, greater or equal to 180 mg/dL, greater than or equal to 153 and less than or equal to 199 mg/dL [22,23,24,25,26,27].

The Control group patients received the usual prenatal care, including nutritional guidance, and the DMG group patients received individualized nutritional therapy, a physical activity program, and glycemic monitoring, as therapies immediately after GDM diagnosis [17,28]. Nutritional therapy consists of a caloric intake program according to pre-gestational body mass index and gestational age at the time of intervention and composition distributed among 40% to 55% of carbohydrates, 15% to 20% of proteins, and 30% to 40% of lipids, in addition to the contribution of at least 28 g of fiber. The physical activity program consists of 30 to 40 min daily, 5 to 7 days a week, of aerobic activity, mainly characterized by walking at moderate intensity [24,25,26].

Glycemic monitoring was performed with four to six points of daily capillary glucosimetry, including fasting, pre-prandial, and post-prandial blood glucose measurements. These were considered good glucose control criteria for inclusion in the GMD group when at least 70% of the capillary glucosimetry analyses were carried out according to international thresholds, namely, fasting blood glucose levels above 63 mg/dL and below 95 mg/dL, 100 mg/dL pre-prandial, 140 mg/dL one hour post-prandial and 120 mg/dL two hours post-prandial [24,25,26].

All pregnant women received at the third trimester of gestation a single oral dose of 20 mg racemic FLX, and serial blood samples were collected at 0, 2, 3, 4, 5, 6, 7, 8, 10, 12, 24, 48, 72, 96, 168, 336, 504 and 672 h after drug administration. Later, on the day of delivery, all pregnant received a new single oral dose of 20 mg racemic FLX at least 2 h before the delivery [28]. Then, one blood sample from a maternal vain, an umbilical cord artery, an umbilical cord vein, and the placental intervillous space, as well as one amniotic fluid sample, were collected from each patient immediately after placental detachment during the delivery.

The blood collection from the placental intervillous space was conducted by the method of Meirelles and Matheus modified by Camelo et al. [29,30]. Briefly, after placental detachment, the retroplacental clot was removed, and the basal plate was closed with the membranes. The placenta was placed inside a plastic bag, allowing it to be lifted and observed. With the chorial plate looking down, the plastic bag was sectioned with scissors, and the chorial plate was perforated with a stylet at a site of the chorial plate in which no fetal vessels were identified. Blood (5 mL) was allowed to drip freely, directly into a collecting tube containing dried 20% ethylenediamine tetraacetic acid (EDTA).

The time, indication, and mode of delivery were decided on according to the care protocols of the institution. All blood samples were centrifuged (1800× *g*, 20 min) and the plasma samples and amniotic fluid were stored at −80 °C until the analysis.

Besides the GDM group, we also used data of FLX and norFLX enantiomer pharmacokinetics of nine healthy pregnant women previously published [17,28] (Control group), who were sampled similarly, as the current study is a continuation of research aiming to add knowledge about the influence of common comorbidities in pregnancy on the pharmacokinetics of FLX and norFLX enantiomers.

### 2.2. Enantioselective Analysis of FLX and NorFLX in Plasma

The concentrations of FLX and NorFLX in plasma samples of maternal vein, umbilical vein and intervillous space were determined by liquid chromatography–tandem mass spectrometry (LC-MS/MS), as previously described [31]. The calibration curves of FLX and NorFLX ranged from 0.04–20 ng/mL to 0.05–10 ng/mL, respectively. The enantiomers were separated using a Chirobiotic V column with a mobile phase consisting of ethanol and 15 mM ammonium acetate (85:15, *v*/*v*). Quantification was performed via LC-MS/MS, achieving a quantitation limit of 0.04 ng/mL for each FLX enantiomer and 0.1 ng/mL for each NorFLX enantiomer in plasma. The inter- and intra-assays of this analytical method presented coefficients of variation and residual standard errors lower than 15%. Quality control samples (low, medium and high analyte concentrations) were analyzed, along with this study samples and calibration standards presenting CV and RSE lower than 15% [31].

### 2.3. Pharmacokinetic and Statistical Analysis

The pharmacokinetics (PK) of FLX and norFLX enantiomers were analyzed individually based on their plasma concentration over time by a mono-compartment model (WinNonLin Classic PK model 3) with first-order absorption without lag-time and first-order elimination in the software WinNonLin version 7.0.0.2535 (Pharsight, Certara, Princeton, NJ, USA).

The AUC_0–∞_ was calculated by the trapezoidal rule; apparent total clearance (CL/F), apparent volume of distribution (Vd/F), maximum plasma concentration (Cmax) and the time to reach Cmax (Tmax) were determined according to the embed WinNonLin equations [32,33].

The influence of GDM on pharmacokinetic parameters and on placental transfer of FLX and norFLX enantiomers was evaluated by nonparametric multiple comparisons with the Kruskal–Wallis’s rank-sum test and with the Dunn–Bonferroni post hoc adjustment assuming significance of 5% and power of 80%. The software R version 3.4.4 (a language and environment for statistical computing) was used to access the statistical summaries of the plasma concentrations and pharmacokinetic parameters of FLX and norFLX enantiomers and figures.

## 3. Results

The anthropometric and clinical data of the pregnant women at the third trimester of pregnancy and their newborns are presented in Table 1 as median and 25–75th percentile. There were no statistical differences in age range, gestational age of inclusion in the study, anthropometric or protein assessment results data between the GDM and control groups.

The pregnant women diagnosed with GDM achieved good glycemic control and had no other comorbidities. No newborns had diseases diagnosed at birth. All newborns had Apgar score greater than 7 at the first and fifth minutes, characterizing well-being at birth (Table 1). All newborns had good postnatal evolution and were medically released at the same time as their mother. None of the placentas showed relevant macroscopic changes.

**Figure 1:**
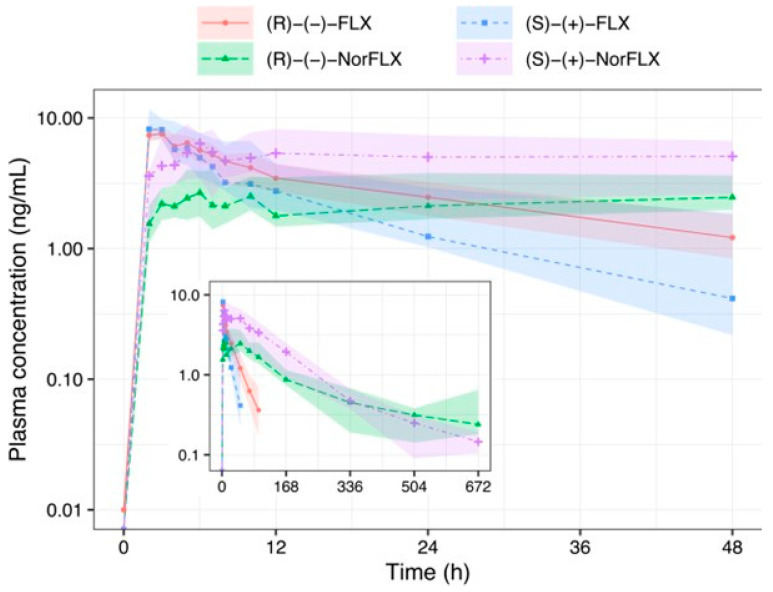
Plasma concentration-over-time curve of fluoxetine and norfluoxetine enantiomers following a single oral dose of 20 mg fluoxetine in pregnant women with gestational diabetes mellitus (GDM group) from 0 to 48 h and from 0 to 672 h (inner panel). Data presented as median and 25th–75th percentiles. (R)-(−)-FLX: (R)-(−)-fluoxetine, (R)-(−)-NorFLX: (R)-(−)-norfluoxetine, (S)-(+)-FLX: (S)-(+)-fluoxetine, (S)-(+)-NorFLX: (S)-(+)-norfluoxetine.

**Figure 2:**
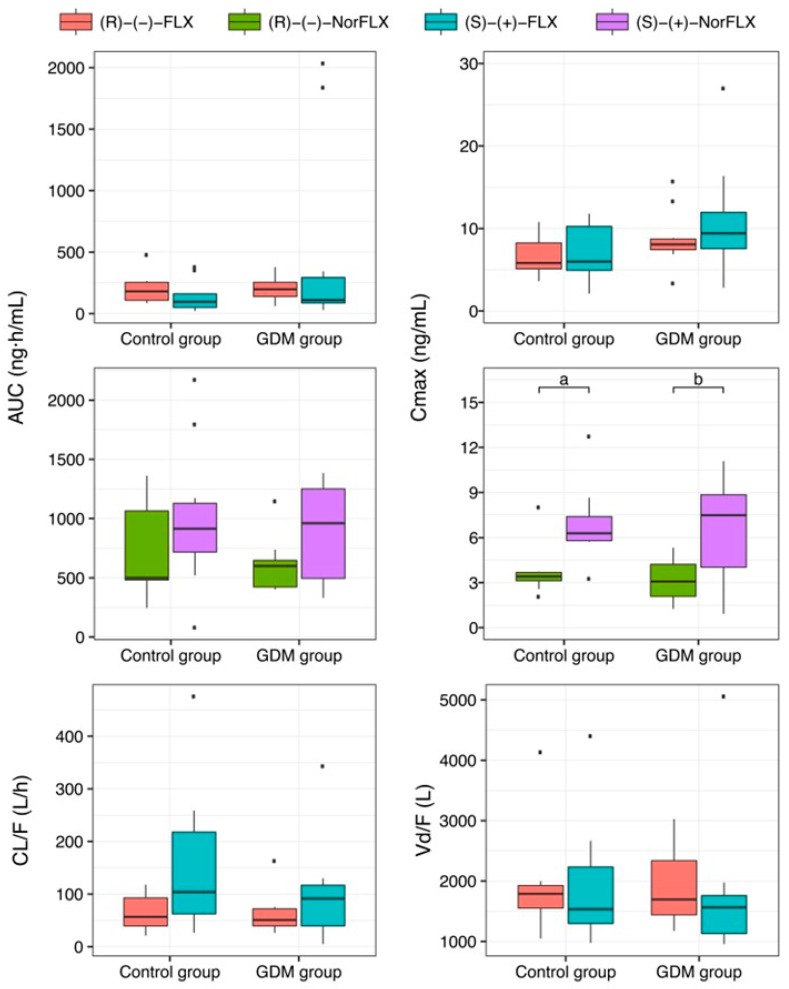
Boxplot of the area under the plasma concentration-over-time curve (AUC) maximum plasma concentration (Cmax). Apparent clearance (CL/F) and apparent volume of distribution (Vd/F) of (R)-(−)-fluoxetine: (R)-(−)-FLX, (R)-(−)-norfluoxetine: (R)-(−)-NorFLX, (S)-(+)-fluoxetine: (S)-(+)-FLX, and (S)-(+)-norfluoxetine: (S)-(+)-NorFLX in healthy pregnant women (Control group) and pregnant women with gestational diabetes mellitus (GDM group) following a single oral dose of fluoxetine 20 mg. a: *p* = 0.031, b: *p* = 0.035. All comparisons between Control group and GDM group were *p* > 0.05. *p*-values assessed by Kruskal–Wallis’s rank-sum test with Dunn–Bonferroni post hoc adjustment.

The following plasma concentration-over-time curves of (R)-(−)-FLX: (R)-(−)-fluoxetine, (R)-(−)-NorFLX: (R)-(−)-norfluoxetine, (S)-(+)-FLX: (S)-(+)-fluoxetine, and (S)-(+)-NorFLX: (S)-(+)-norfluoxetine after a single oral dose of FLX 20 mg in the GDM group are presented in Figure 1 from 0 to 48 h and from 0 to 672 h, while the pharmacokinetic parameters are shown in Table 2 and Figure 2. The data indicate that the pharmacokinetic parameters are comparable between the GDM group and the Control group, showing no significant differences [17] (Figure 2).

**Figure 3:**
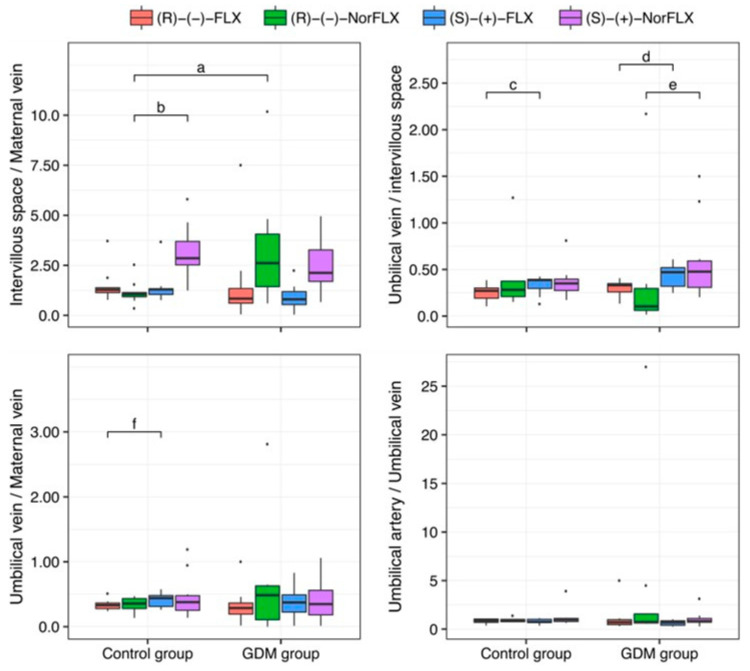
Box-plot of placental transfer ratios of fluoxetine and norfluoxetine enantiomers in healthy pregnant women (Control group) and pregnant women with gestational diabetes mellitus (GDM) following a single oral dose of fluoxetine 20 mg. (R)-(−)-FLX: (R)-(−)-fluoxetine. (R)-(−)-NorFLX: (R)-(−)-norfluoxetine. (S)-(+)-FLX: (S)-(+)-fluoxetine. (S)-(+)-NorFLX: (S)-(+)-norfluoxetine. *p*-values assessed by Kruskal–Wallis’s rank-sum test with Dunn–Bonferroni post hoc adjustment. a: *p* = 0.000, b: *p* = 0.023, c: *p* = 0.049, d: *p* = 0.048, e: *p* = 0.017, f: *p* = 0.049.

The placental transfer data obtained from the GDM group compared to pregnant women in the Control group are presented in Table 3. Statistical differences were observed in the EIV/VM ratios for R-(−)-NorFLX and in the VU/EIV ratios for R-(−)-NorFLX when comparing the GDM group to the Control group [17]. Except for the intervillous space/maternal vein ratio, the placental transfer does not differ between the Control [17] and GDM groups (Figure 3).

## 4. Discussion

The pharmacokinetics and transplacental transfer of FLX and norFLX enantiomers were evaluated in 10 pregnant women with GDM (GDM group) and the values were compared with data of 9 healthy pregnant women (Control group) previously described by the research group [17]. The anthropometric and clinical data do not differ between the groups (Table 1). It is noteworthy that the GDM pregnant women had been diagnosed and, during pregnancy, were on a non-drug GDM treatment, achieving normal glycosylated hemoglobin and blood glucose levels before they were included in the clinical trial.

The plasma exposure (AUC_0-∞_ values) of the (R)-(−)-FLX enantiomer did not differ from the plasma exposure of the (S)-(+)-FLX enantiomer in both GDM and Control [17] groups following a single oral dose of 20 mg FLX (Table 2). However, Kim et al. (2006) [34] report higher plasma exposure at steady-state of the (S)-(+)-FLX enantiomer with (S)-(+)-FLX/(R)-(−)-FLX AUC ratios close to 2 in healthy pregnant women treated with 20 mg FLX daily. Considering that only CYP2D6 is involved in the metabolism of (S)-(+)-FLX, whereas the metabolism of its antipode is catalyzed by CYP2C9 and CYP2D6, and considering that both FLX and norFLX are CYP2D6 inhibitors, the repeated administration of FLX inhibits the metabolism of the (S)-(+)-FLX enantiomer, explaining its higher plasma exposure at the repeated administration [35]. However, in the present study, the participants received a single FLX dose, and the CYP2D6 isoform was probably not inhibited, with the consequent similar plasma exposure of both FLX enantiomers.

The apparent total clearance and apparent volume of distribution of R-(−)-FLX and S-(+)-FLX did not differ between the Control [17] and GDM groups (Table 2). The AUC_0-∞_ ratios between (R)-(−)-norFLX/(R)-(−)-FLX and between (S)-(+)-norFLX/(S)-(+)-FLX (Table 2) were not different between Control [17] and GDM groups (*p* > 0.05, obtained by Kruskal–Wallis’s rank-sum test with the Dunn–Bonferroni post hoc test), as well. Therefore, the GDM did not influence the pharmacokinetics of FLX enantiomers and the formation of the metabolite norFLX in pregnant women with good glycemic control.

Diabetes mellitus can change intestinal absorption, distribution and metabolism of drugs, due to the cellular metabolism modification induced by glucose accumulation, long-term glycated biomolecules or glycation of drugs and their metabolites [18]; however, this feature of metabolic modification is not present in glycemic-controlled diabetes, as in the case of the present-study GDM patients with good glycemic control. Similarly, the pharmacokinetics parameters of nifedipine [19] and metoprolol [20] are not changed in pregnant women with good glycemic control investigated in the third trimester of gestation. Therefore, the present and previous findings suggest GMD [19,20] and diabetes mellitus [36,37] patients, who can achieve glucose levels at threshold values, regardless of the treatment, apparently have the same chance as a non-diabetic-diagnosed subject of presenting PK drug changes.

As previously reported for healthy pregnant women [17], in the GDM group the Cmax values of the (S)-(+)-nor-FLX (7.48 ng/mL) were also higher than those observed for the (R)-(−)-norFLX (3.08 ng/mL) (Table 2 and Figure 2), probably because in both healthy and GDM pregnant women, the (S)-(+)-FLX enantiomer with metabolism dependent on CYP2D6 is metabolized to NorFLX to a higher extent than the (R)-(−)-FLX enantiomer, in which the metabolism is dependent on other CYP isoforms. Although statistically not significant, the higher AUC values of (S)-(+)-NorFLX and (R)-(+)-FLX, when compared to their antipodes (Table 2, Figure 2), allows us to suppose that, in both healthy and GDM pregnant women, the (S)-(+)-FLX enantiomer is metabolized to NorFLX to a higher extent than (R)-(−)-FLX.

Regarding placental transfer, the umbilical vein/maternal vein plasma-concentration ratios were nearly 0.3 for both enantiomers of FLX and NorFLX, allowing us to infer a low placental transfer in the GDM group, but also in the Control group [17] (Table 3, Figure 3). The umbilical artery/umbilical vein plasma-concentration ratios were nearly 0.7 for both enantiomers of FLX and NorFLX in all investigated pregnant women diagnosed or not with GDM (Table 3), indicating the absence of or minimum fetal metabolism (Figure 3).

The present study has some noticeable limitations, such as the lack of CYP2D6 genotype or phenotype and unbound plasma concentrations of FLX and norFLX. The absence of GDM patients with hyperglycemia does not allow the present findings to lead to implications about the poorly controlled GDM condition. Moreover, the study sampled the BMI and other anthropometric data during the third trimester of pregnancy and puerperium, meaning that pre-pregnancy anthropometric values were not found [38], and the lack of data regarding the relationship between FLX plasma exposure and clinical outcome is another limitation. As expected, none of the placentas showed macroscopic changes, because the patients went through good glycemic control after GDM diagnosis; however, we did not search for changes at the histopathological microscopic level that, according to previous reports, may occur in GDM [39,40,41].

## 5. Conclusions

In summary, the GDM does not influence the pharmacokinetics of FLX and NorFLX enantiomers following a single oral dose of 20 mg racemic FLX in pregnant women with good glycemic control. In addition, the placental transfer (umbilical vein/maternal vein ratios) of FLX and NorFLX enantiomers is low in these pregnant women. The absence of GDM patients with hyperglycemia in the present study opens the possibility of research focusing on GDM patients with poor glycemic control.

## Figures and Tables

**Table 1:** Anthropometric and clinical data of the investigated pregnant women and their newborns.

Parameter	Control Group [17] (n = 9)	GDM Group (n = 10)
Age (years) #	30.5 (22.5–35.5)	32.00 (29.50–37.25)
Gestational age (days) #	236.5 (224.75–243.50)	224.00 (217.75–231.25)
Maternal weight (kg) #	72.65 (68.67–76.40)	81.67 (66.25–87.72)
Maternal height (m) #	1.60 (1.54–1.67)	1.59 (1.56–1.65)
BMI (kg/m^2^) #	29.00 (25.00–32.00)	30.00 (28.00–35.00)
Newborn weight (g) *	3220 (3180–3305)	3255 (2975–3477)
Newborn height (cm) *	49.50 (48.00–50.00)	48.75 (47.63–49.88)
Placenta weight (g) *	500 (430–600)	507.50 (505.00–545.00)
Apgar index *	9.50 (9.00–10.00)	9.00 (8.00–9.00)
Total proteins (g/dL) #	6.40 (6.20–6.60)	6.10 (5.80–6.40)
Albumin (g/dL) #	3.90 (3.82–3.92)	3.65 (3.60–3.80)
α1-acid glycoprotein (mg/dL) #	56.00 (49.05–61.20)	50.15 (45.08–62.80)
Glycosylated hemoglobin (%) #	5.55 (5.10–6.03)	5.15 (4.57–5.25)
Glycemia (mg/dL) #	87.00 (85.00–91.50)	74.00 (72.00–84.00)

Data presented as median and 25–75th percentiles. # Data collected at the inclusion of the patient in the study (third trimester of gestational age). * Data collected at the delivery. No differences were found between Control [17] and GDM groups (no *p* < 0.05, Wilcoxon test).

**Table 2:** Pharmacokinetics parameters of fluoxetine (FLX) and norfluoxetine (NorFLX) enantiomers following a single oral dose of fluoxetine 20 mg in healthy pregnant women (Control group) [17] and pregnant women with gestational diabetes mellitus (GDM group).

	Control Group (n = 9) [17]				GDM Group (n = 10)			
	R-(−)-FLX	S-(+)-FLX	R-(−)-norFLX	S-(+)-norFLX	R-(−)-FLX	S-(+)-FLX	R-(−)-norFLX	S-(+)-norFLX
Cmax (ng/mL)	5.94 (5.14–9.16)	6.05 (4.04–11.46)	3.41 ** (3.13–3.68)	6.29 ** (5.80–7.39)	8.07 (7.44–8.72)	9.42 (7.56–11.96)	3.08 ** (2.08–4.22)	7.48 ** (4.03–8.84)
tmax (h)	4.41 (2.39–5.26)	2.35 (1.62–3.59)	20.60 * (14.74–23.10)	13.66 (7.69–20.81)	2.37 (1.38–2.81)	1.72 (0.91–2.49)	10.95 * (9.61–11.71)	10.68 (8.16–25.93)
Ka (h^−1^)	0.33 (0.24–0.55)	0.37 (0.18–0.52)	--	--	0.38 (0.24–0.58)	0.48 (0.38–0.55)	--	--
AUC_0-∞_ (ng∙h/mL)	209.20 (113.90–263.00)	96.22 (49.61–160.94)	500.33 (482.78–1063.96)	942.70 (802.81–1172.15)	197.93 (139.27–254.41)	109.62 (85.75–293.70)	600.39 (423.08–648.18)	960.83 (495.88–1250.82)
CL/F (L/h)	56.65 (39.45–92.71)	103.95 (62.28–217.94)	---	---	50.78 (39.58–71.96)	91.63 (39.44–116.96)	---	---
α (h^−1^)	0.18 (0.11–0.30)	0.16 (0.11–0.36)	---	---	0.375 (0.237–0.579)	0.483 (0.382–0.546)	---	---
Vd/F (L)	1787.81 (1552.43–1925.23)	1533.18 (1296.74–2233.72)	---	---	1695.15 (1440.18–2338.19)	1564.90 (1130.57–1760.14)	---	---
β (h^−1^)	0.03 (0.02–0.04)	0.04 (0.03–0.07)	0.007 0.004–0.009	0.008 0.005–0.011	0.026 (0.023–0.034)	0.039 (0.027–0.053)	0.005 (0.004–0.008)	0.008 (0.006–0.009)
t1/2 (h)	26.74 (18.64–32.29)	17.21 (11.97–18.76)	99.55 (82.87–143.85)	83.48 (72.30–131.57)	27.06 (20.79–29.76)	18.41 (13.06–25.81)	152.04 (83.15–201.08)	90.55 (75.13–118.74)
	**R-(** **−)**		**S-(+)**		**R-(** **−)**		**S-(+)**	
AUC_0-∞_ $\left(\frac{\mathrm{norFLX}}{\mathrm{FLX}}\right)$	2.93 (2.31–5.19)		8.78 (6.07–16.17)		3.25 (1.98–5.01)		8.92 (2.66–14.24)	

Data are presented as median and 25–75th percentile. (R)-(−)-FLX: (R)-(−)-fluoxetine. (R)-(−)-norFLX: (R)-(−)-norfluoxetine. (S)-(+)-FLX: (S)-(+)-fluoxetine. (S)-(+)-norFLX: (S)-(+)-norfluoxetine. All comparisons between Control group and GDM group are *p* > 0.05. * *p* <0.05, Control group vs. GDM group, Kruskal–Wallis’s rank-sum test with Dunn–Bonferroni post hoc test; ** *p* < 0.05, R-(-) vs. S-(+), Kruskal–Wallis’s rank-sum test with Dunn–Bonferroni post hoc test.

**Table 3:** Placental transfer ratios of fluoxetine (FLX) and norfluoxetine (norFLX) in healthy pregnant women (Control group) [17] and pregnant women with gestational diabetes mellitus (GDM group) enantiomers, following a single oral dose of fluoxetine 20 mg.

	Control Group (n = 9) [17]				GDM Group (n = 10)			
	R-(−)-FLX	S-(+)-FLX	R-(−)-norFLX	S-(+)-norFLX	R-(−)-FLX	S-(+)-FLX	R-(−)-norFLX	S-(+)-norFLX
UA/UV	0.86 (0.70–1.04)	0.74 (0.71–1.01)	0.87 (0.76–1.01)	1.00 (0.79–1.10)	0.71 (0.46–0.98)	0.68 (0.40–0.86)	0.73 (0.63–1.57)	0.82 (0.71–1.10)
IS/MV	1.28 (1.14–1.38)	1.30 (1.05–1.33)	1.06 * (0.93–1.14)	2.86 ** (2.52–3.69)	0.84 (0.61–1.34)	0.80 (0.54–1.19)	2.61 * (1.43–4.06)	2.12 (1.70–3.27)
UV/IS	0.27 (0.19–0.30)	0.39 ** (0.30–0.39)	0.28 (0.21–0.38)	0.35 ** (0.27–0.40)	0.33 (0.26–0.35)	0.47 (0.32–0.52)	0.10 (0.06–0.30)	0.48 ** (0.31–0.59)
UV/MV	0.33 (0.28–0.36)	0.44 ** (0.30–0.48)	0.36 (0.28–0.43)	0.38 (0.25–0.48)	0.29 (0.19–0.36)	0.37 (0.22–0.49)	0.48 (0.11–0.63)	0.35 (0.18–0.56)
Latency (minutes)	198.00 (155.00–239.00)				159.00 (138.00–212.00)			

Data expressed as median and 25–75th percentiles. UA/UV: umbilical artery/umbilical vein; IS/MV: intervillous space/maternal vein; UV/IS: umbilical vein/intervillous space; UV/MV: umbilical vein/maternal vein. (R)-(−)-FLX: (R)-(−)-fluoxetine. (R)-(−)-norFLX: (R)-(−)-norfluoxetine. (S)-(+)-FLX: (S)-(+)-fluoxetine. (S)-(+)-NorFLX: (S)-(+)-norfluoxetine. * *p* < 0.05, Control group vs. GDM group, Kruskal–Wallis’s rank-sum test with Dunn–Bonferroni post hoc test; ** *p* < 0.05, R-() vs. S-(+), Kruskal–Wallis’s rank-sum test with Dunn–Bonferroni post hoc test.

## Data Availability

Original data are available from the corresponding author upon request.
